# Nap sleep spindle correlates of intelligence

**DOI:** 10.1038/srep17159

**Published:** 2015-11-26

**Authors:** Péter P. Ujma, Róbert Bódizs, Ferenc Gombos, Johannes Stintzing, Boris N. Konrad, Lisa Genzel, Axel Steiger, Martin Dresler

**Affiliations:** 1 Institute of Behavioural Sciences, Semmelweis University, H-1089 Budapest, Hungary; 2 National Institute of Clinical Neuroscience, Epilepsy Centrum, Department of Neurology, H-1145 Budapest, Hungary; 3 Department of General Psychology, Pázmány Péter Catholic University, H-1088 Budapest, Hungary; 4 Max Planck Institute of Psychiatry, 80804 Munich, Germany; 5 Donders Institute for Brain, Cognition and Behaviour, Radboud University Medical Centre, 6525 EN Nijmegen, The Netherlands; 6 Centre for Cognitive and Neural Systems, University of Edinburgh, EH8 9JZ Edinburgh, UK

## Abstract

Sleep spindles are thalamocortical oscillations in non-rapid eye movement (NREM) sleep, that play an important role in sleep-related neuroplasticity and offline information processing. Several studies with full-night sleep recordings have reported a positive association between sleep spindles and fluid intelligence scores, however more recently it has been shown that only few sleep spindle measures correlate with intelligence in females, and none in males. Sleep spindle regulation underlies a circadian rhythm, however the association between spindles and intelligence has not been investigated in daytime nap sleep so far. In a sample of 86 healthy male human subjects, we investigated the correlation between fluid intelligence and sleep spindle parameters in an afternoon nap of 100 minutes. Mean sleep spindle length, amplitude and density were computed for each subject and for each derivation for both slow and fast spindles. A positive association was found between intelligence and slow spindle duration, but not any other sleep spindle parameter. As a positive correlation between intelligence and slow sleep spindle duration in full-night polysomnography has only been reported in females but not males, our results suggest that the association between intelligence and sleep spindles is more complex than previously assumed.

Sleep spindles are thalamocortical oscillations^1^,^2^ emerging during NREM sleep with the physiological potential to facilitate neuroplasticity^3^,^4^,^5^. Sleep spindle characteristics such as spindle density, frequency or amplitude are trait-like individual characteristics with genetic and anatomical underpinnings^6^,^7^,^8^. The hypothesis that trait-like electrophysiological characteristics with a function so closely related to cognition may be related to individual cognitive performance were corroborated in early studies which revealed a positive correlation between several sleep spindle parameters and intelligence^9^,^10^,^11^,^12^,^13^,^14^. However, a recent review suggested that reports on a positive association between spindles and intelligence are often inconsistent and mostly rely on small sample sizes, and that positive findings might be strongly overrepresented in the literature due to publication bias^15^. In addition, cognitive performance has been shown to correlate with anatomical properties of the brain in a strongly sex-dependent manner^16^,^17^, characterized by a positive correlation with white matter morphometric data in females, but less so in males. Sleep spindle amplitude also heavily relies on the morphology of thalamocortical white matter tracts^18^. In line with these observations recent findings have confirmed that sleep spindle parameters correlate with intelligence mainly in females, and less so in males^15^,^19^.

In the present study, we aimed to corroborate and extend these findings by investigating the correlates of intelligence in a large sample of male subjects recorded during afternoon naps. Nap sleep spindles are typically considered as representative of night spindle activity as they are similarly associated with neuroplasticity processes. However, sleep spindles are also modulated by circadian regulation^20^. We hypothesized that – in line with recent findings – no significant correlation between intelligence and sleep spindle parameters would be observed in afternoon naps. Results inconsistent with this hypothesis would suggest that the spindle-intelligence association might be different for night sleep and afternoon naps.

## Materials and Methods

### Participants and Instructions

86 male subjects were recruited at local universities through flyers and email lists and were paid for their participation. Exclusion criteria as assessed in a screening interview included a history of sleep disorders (assessed also via Pittsburgh Sleep Quality Index^21^, drug abuse, or psychiatric or neurological diseases; further shift work, transmeridian flights within the past month, regular daytime naps, extreme chronotype (assessed via Morningness-Evenings-Questionnaire^22^, or regular consumption of more than 2 cups of coffee or 5 cigarettes per day. 7 subjects were excluded from the study due to their failure to produce at least one epoch of N2 sleep. The mean age for the remaining 79 subjects was 23.29 years (SD 2.63 years, range 18–30 years). Experimental procedures were approved by the ethics committee of the University of Munich, participants gave written informed consent, and all procedures were carried out in accordance with the approved guidelines.

Participants were instructed to follow a regular sleep pattern in the week preceding the sleep recording, which was controlled by sleep diaries. On the morning of the sleep recording, participants were instructed to get up not after 7 a.m. to increase probability of falling asleep during sleep recording. Participants arrived at 13:00 hours at the sleep laboratory. After electrode placement, participants had to complete the German version of Culture Fair Test (CFT 20-R)^23^, a non-verbal test of fluid intelligence with a high g-loading^24^. The mean IQ was 116.4 (SD 15.1, range 80–143).

They further completed questionnaires on handedness, vocabulary, and self-assessed creativity (not analyzed here), however did not engage in any memory-related activity to avoid respective confounding effects on sleep spindle activity.

### Data Acquisition

After EEG application and behavioral testing, during a 100 minutes lights off period participants underwent polysomnography using a digital recorder (Comlab 32 Digital Sleep Lab, Brainlab V 3.3 Software, Schwarzer GmbH, Munich, Germany) with a sampling rate of 250 Hz with 6 EEG electrodes (F3, F4, C3, C4, O1, O2, all referenced to the contralateral mastoids), EMG, ECG and EOG. Polysomnography recordings were scored by 30 second epochs according to standard criteria^25^.

### Data Analysis

Polysomnography recordings were analyzed using a methodology similar to our recent studies^15^,^19^. N2 and N3 sleep epochs were subjected to automated sleep spindle analysis using the Individual Adjustment Method (IAM)^26^,^27^: High-resolution (bin width: 0.0625 Hz) average amplitude spectrum (with zero-padding) of the signal of EEG electrodes and the second-order derivatives of a down-sampled (0.25 Hz) average amplitude spectrum were computed. Two spectral peaks corresponding to slow and fast spindles were identified based on the zero-crossings of the average of the second order derivatives of the spectra of all available EEG derivations. The edges of these spectral peaks were defined on the frequency scale of the high resolution spectra as the individual slow and fast spindle frequency ranges, respectively. EEG data was filtered for these individual sleep spindle frequency ranges, and a sleep spindle was detected wherever the envelope of the filtered signal exceeded a derivation-specific amplitude criterion for at least 0.5 seconds. The amplitude criterion was defined as the mean of the high resolution amplitude spectral values of NREM sleep EEG of the given electrode at the boundaries of the individual sleep spindle frequency range, multiplied by the number of bins within the individual frequency range. The individual mean length and mean maximum amplitude (defined by the mean maximum of the envelopes of filtered EEG signals over the detected spindles) as well as sleep spindle density (number of sleep spindles/ minute) was computed for each subject and each derivation for both slow and fast spindles.

We computed Pearson’s point-moment correlations between IQ scores and individual slow and fast spindle parameters (frequency, density, duration and amplitude). In order to control for multiple comparisons we implemented the Benjamini-Hochberg procedure of false discovery rate (FDR) correction^28^.

## Results

**Figure 1:**
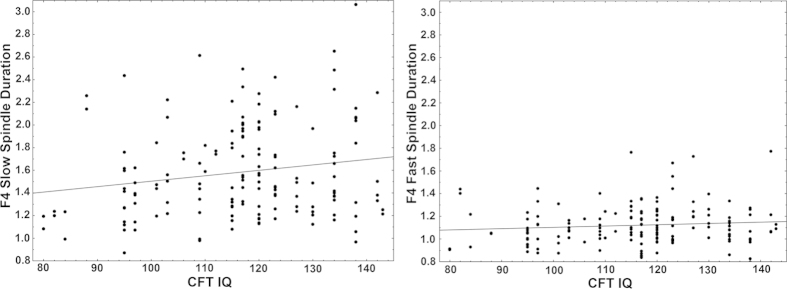
Scatterplots illustrating the correlation between slow (left panel) and fast (right panel) spindle duration (axis y) and CFT IQ score (axis x) on the electrode F4 where the effect was found to be the strongest. While the correlation is only significant in case of slow spindle duration, the two correlation coefficients are not statistically different from each other (Fisher’s z = 3.12, p > 0.1).

We found a positive correlation between intelligence and slow spindle duration (statistically significant on all electrodes, significant after FDR correction). A tendency for a positive correlation between intelligence and slow spindle density on some electrodes was also seen, which however was not significant after FDR correction. No correlation between intelligence and either sleep spindle amplitude or frequency was found, and no correlation between intelligence and any of the fast spindle parameters was seen either. However, correlations with intelligence did not differ significantly between slow and fast spindles. Intelligence did not correlate significantly with any measures of sleep macrostructure. Our results are summarized in Table 1; the strongest results are illustrated as a scatterplot on Fig. 1. Consistent with the narrow age range of our subjects, our results did not change significantly if partial correlations (controlling for age) were calculated.

**Figure 2:**
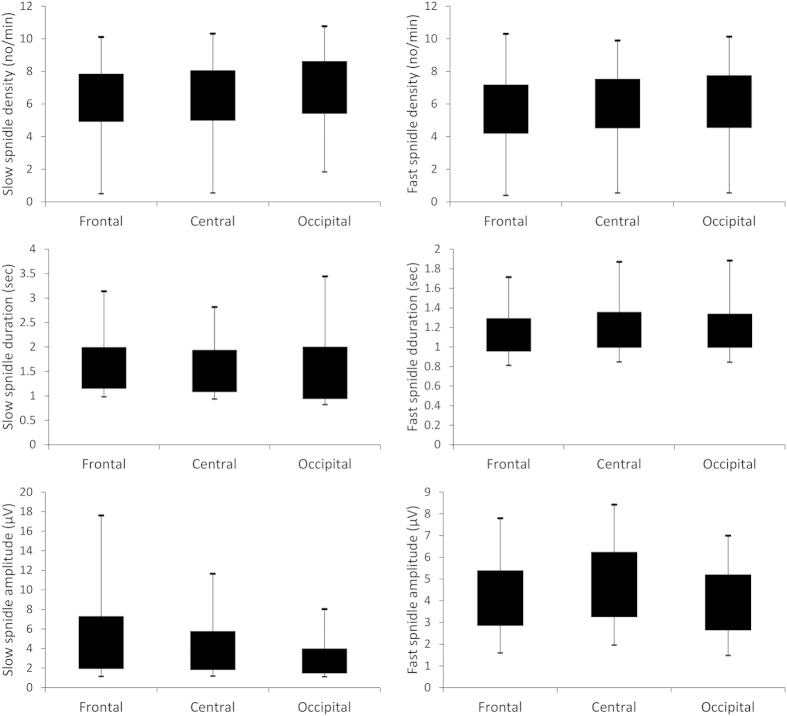
Nap sleep spindle parameters at frontal, central and occipital electrodes.

In order to assess the similarity between nap and night spindle parameters, individual sleep spindle parameter averages obtained on the electrode F4 were compared with the average parameters obtained from the comparable sample of 57 male subjects under 30 years old participating in our earlier night sleep study^15^, analyzed with the same detection algorithm. Both slow and fast spindle density was higher in night sleep (p < 0.001 in both cases). Slow spindle duration and mean amplitude were not significantly different in naps and night sleep. Fast spindle duration was longer in naps (p < 0.05) while mean amplitude was higher in night sleep (p < 0.001). Both slow and fast spindle middle frequency was lower in night sleep (p < 0.001 in both cases). This is in line with previous comparisons of day and night spindles^29^, which also found higher frequency and duration (on a frontal electrode), but lower density and amplitude in naps (albeit the density effect was only significant on a parietal electrode and the amplitude effect remained a tendency on both). Sleep macrostructure and spindle parameters are reported in Tables 2, 3, 4, 5, 6; topographic differences in nap sleep spindle parameters are represented on Fig. 2.

## Discussion

In a large sample of only male subjects, we did not find any correlation between intelligence and fast spindle parameters, and no significant correlation between intelligence and slow spindle density or amplitude. This confirms recent full-night sleep recordings with similar null-findings^15^. In particular the absence of a positive correlation between fast spindle amplitude and intelligence in males is in line with previous night sleep studies revealing such a correlation only in females both in case of adolescents^19^ and adults^15^. In contrast to null-findings in full-night sleep recordings, however, we found a positive correlation between intelligence and slow spindle duration in our male sample, which had previously been reported for females only^15^.

Delta and theta power in naps appear to generally contribute to the same homeostatic processes as night sleep^30^, but sleep spindles are particularly dependent on circadian regulation^20^. The main differences between day and night spindles have been observed for spindle frequency and density^31^, in line with the melatonin-dependent circadian regulation of spindles^20^,^29^. However, less prominent day-night differences in spindle duration and amplitude have been reported, not always reaching statistical significance^29^,^31^. Several reports suggest that day sleep spindles recorded during nap sleep periods are indeed involved in neural plasticity processes as night spindles are, supporting the consolidation of memories in both children^32^ and adults^33^. Sleep spindles recorded in afternoon naps could thus be considered as representative of night spindle activity, and therefore good candidate markers of IQ. In the light of our current results, nap spindles might even be a more sensitive marker of cognitive processing than night sleep spindles as evidenced by a positive association between intelligence and slow spindle duration, previously only seen in females^15^. Sleep spindles preferentially occur during the up-states of cortical slow oscillations^1^, and durations most likely reflect the length of such up-states. Previous reports^34^ found a positive correlation between slow wave upstate length and memory consolidation, as well as the coupling strength of sleep spindles to slow oscillations and intelligence^14^, suggesting that the coupling of these oscillations may be functionally important for cognitive functioning and intelligence.

It must be noted, however, that i) the correlations we found in young napping males were still weaker in effect size than what we previously found in a much more heterogeneous female subsample^15^ and ii) we were unable to reproduce this finding in a re-analysis of 57 night sleep EEG recordings from our previous study^15^ including only male subjects below 30 years of age: in this subsample, no correlation between IQ scores and slow spindle duration was seen (see Table 2). Therefore, while some significant positive correlations were found in our male-only napping sample, the weak effect size of our positive findings about the correlation between intelligence and nap slow spindle duration do not firmly ascertain whether such an association in males is specific for nap sleep. Also, since there was a lack of a significant difference between slow and fast spindle correlation coefficients (see Fig. 2), it cannot be claimed with certainty that this association is specific for slow spindles.

Overall, our results confirm that sleep spindle amplitude is not correlated with intelligence in males, supporting the view of a sexual dimorphism of the neural mechanisms behind intelligence^16^,^17^ with a triangular relationship between white matter morphology, sleep spindle amplitude and intelligence only in females^15^,^17^,^18^. It further confirms our recent null findings regarding other spindle parameters, which had been reported before in studies with smaller sample sizes^15^. Since many previous studies reported on a large number of sleep spindle variables with little consistency in their methodology and because null findings are less likely to be published^15^, the spindle-intelligence association might have been overestimated due to publication bias. Further research into the possible relationship between sleep spindling and intelligence is required, including possible interactions with circadian rhythms and the inclusion of female participants in order to reproduce the positive correlation between sleep spindle amplitude and intelligence also in nap sleep. A direct study of the association between nap and night spindle activity and intelligence in the same subjects would help clarify whether some or all spindle correlates of intelligence are specific for nap vs. full-night sleep.

## Additional Information

**How to cite this article**: Ujma, P. P. *et al*. Nap sleep spindle correlates of intelligence. *Sci. Rep*. **5**, 17159; doi: 10.1038/srep17159 (2015).

## Figures and Tables

**Table 1:** Correlations between sleep spindle parameters and intelligence in nap sleep recordings of 79 healthy young male subjects.

	Nap slow spindles						Nap fast spindles					
	Density		Duration		Amplitude		Density		Duration		Amplitude	
	*r*	*p*	*r*	*p*	*r*	*p*	*r*	*p*	*r*	*p*	*r*	*p*
F3	0.178	0.117	0.299*	0.007	−0.015	0.899	0.072	0.530	0.109	0.337	0.126	0.269
F4	0.169	0.137	0.312*	0.005	−0.030	0.794	0.033	0.774	0.112	0.326	0.098	0.390
C3	0.245	0.029	0.307*	0.006	−0.020	0.862	0.076	0.505	0.107	0.349	0.131	0.251
C4	0.234	0.038	0.286*	0.011	−0.045	0.694	0.036	0.754	0.139	0.223	0.105	0.355
O1	0.183	0.107	0.251*	0.026	−0.044	0.704	0.102	0.373	0.098	0.390	0.078	0.497
O2	0.241	0.032	0.294*	0.008	−0.072	0.531	0.164	0.149	0.090	0.430	0.039	0.736

Correlation coefficients which remain significant after correcting for multiple testing are marked by an asterisk.

**Table 2:** Correlations between sleep spindle parameters and intelligence in full-night sleep recordings of 57 healthy young male subjects.

	Night slow spindles						Night fast spindles					
	Density		Duration		Amplitude		Density		Duration		Amplitude	
	*r*	*p*	*r*	*p*	*r*	*p*	*r*	*p*	*r*	*p*	*r*	*p*
F3	0.087	0.518	0.002	0.989	0.032	0.814	−0.170	0.206	−0.009	0.946	0.053	0.698
F4	−0.020	0.884	−0.016	0.906	0.024	0.860	−0.160	0.234	0.054	0.690	0.073	0.589
C3	0.144	0.284	−0.017	0.902	0.046	0.734	−0.226	0.091	−0.014	0.917	0.101	0.453
C4	0.053	0.696	−0.035	0.796	0.005	0.972	−0.180	0.181	0.023	0.867	0.126	0.349
O1	0.123	0.363	−0.059	0.666	0.017	0.901	−0.269	0.043	−0.029	0.833	0.076	0.574
O2	0.127	0.345	−0.057	0.674	0.017	0.899	−0.281	0.034	−0.029	0.830	0.037	0.784

No correlation coefficients remained significant after correcting for multiple comparisons.

**Table 3:** Nap sleep EEG macrostructure given in minutes.

	Mean	Std. Dev.	Minimum	Maximum
Sleep duration	70.39	19.27	21.5	103.5
Wake duration	24.17	19.67	0	70.5
S1 duration	23.18	13.48	1.5	72.0
S2 duration	30.05	14.42	4.0	71.5
SWS duration	11.43	12.07	0	44.0
REM duration	5.73	6.60	0	24.0
Sleep latency (first sleep)	12.24	6.65	4.0	38.5
REM latency (from first sleep)	59.23	22.68	3.5	89.0

**Table 4:** Full-night sleep EEG macrostructure given in minutes.

	Mean	Std. Dev.	Minimum	Maximum
Sleep duration	432.50	31.49	323.3	474.7
Wake duration	42.01	29.73	0.0	157.7
S1 duration	16.95	12.93	0.7	53.0
S2 duration	229.28	31.71	156.3	296.0
SWS duration	83.45	28.61	2.0	172.0
REM duration	102.82	24.84	51.7	147.7
Sleep latency (first sleep)	24.28	19.80	0.0	98.7
REM latency (from first sleep)	88.65	31.68	9.7	176.7

**Table 5:** Nap sleep spindle parameters.

		Nap slow spindles				Nap fast spindles			
		*Mean*	*StD*	*Minimum*	*Maximum*	*Mean*	*StD*	*Minimum*	*Maximum*
	Frequency (low)	11.512	0.732	9.576	13.008	13.505	0.432	12.588	14.422
	Frequency (high)	12.335	0.691	10.461	13.839	14.560	0.459	13.629	15.745
	Frequency (middle)	11.924	0.701	10.078	13.424	14.052	0.435	13.109	15.056
**C3**	Density	6.468	1.528	0.396	10.678	6.034	1.479	0.593	9.581
	Duration	1.511	0.432	0.925	2.899	1.173	0.180	0.843	1.891
	Mean amplitude	3.789	1.897	1.142	11.021	4.664	1.443	1.848	8.284
	Maximum amplitude	4.025	2.014	1.223	12.048	4.951	1.511	1.957	8.660
**C4**	Density	6.581	1.537	0.693	9.957	6.016	1.520	0.495	10.210
	Duration	1.507	0.421	0.945	2.733	1.177	0.182	0.850	1.849
	Mean amplitude	3.795	2.020	1.232	12.268	4.829	1.532	2.072	8.559
	Maximum amplitude	4.040	2.129	1.363	13.157	5.123	1.587	2.197	8.911
**F3**	Density	6.335	1.423	0.297	10.215	5.694	1.424	0.297	9.840
	Duration	1.577	0.430	0.966	3.214	1.123	0.168	0.799	1.700
	Mean amplitude	4.572	2.609	1.110	17.421	4.020	1.238	1.551	8.218
	Maximum amplitude	4.869	2.729	1.199	17.855	4.314	1.300	1.663	8.496
**F4**	Density	6.428	1.493	0.693	10.003	5.674	1.555	0.495	10.764
	Duration	1.566	0.414	0.992	3.064	1.124	0.168	0.825	1.728
	Mean amplitude	4.655	2.735	1.184	17.792	4.222	1.291	1.640	7.374
	Maximum amplitude	4.967	2.857	1.253	18.184	4.531	1.367	1.760	7.849
**O1**	Density	7.013	1.624	2.177	10.620	6.120	1.632	0.594	9.761
	Duration	1.464	0.539	0.813	3.680	1.163	0.182	0.821	1.985
	Mean amplitude	2.736	1.257	1.046	8.390	3.995	1.275	1.620	7.017
	Maximum amplitude	2.944	1.387	1.068	9.356	4.229	1.327	1.787	7.410
**O2**	Density	7.025	1.580	1.485	10.929	6.173	1.570	0.495	10.505
	Duration	1.475	0.520	0.829	3.207	1.170	0.162	0.868	1.782
	Mean amplitude	2.725	1.241	1.182	7.695	3.852	1.271	1.343	6.975
	Maximum amplitude	2.935	1.361	1.211	8.691	4.109	1.319	1.438	7.320

**Table 6:** Full-night sleep spindle parameters.

		Night slow spindles				Night fast spindles			
		*Mean*	*StD*	*Minimum*	*Maximum*	*Mean*	*StD*	*Minimum*	*Maximum*
	Frequency (low)	10.901	0.594	9.524	12.194	12.820	0.495	11.822	13.911
	Frequency (high)	11.803	0.562	10.280	12.995	14.064	0.577	13.040	15.335
	Frequency (middle)	11.352	0.557	9.902	12.480	13.442	0.532	12.498	14.559
**C3**	Density	7.002	1.220	3.094	9.370	7.559	0.808	5.471	9.116
	Duration	1.412	0.475	0.804	3.176	1.133	0.141	0.863	1.418
	Mean amplitude	3.271	9.480	1.457	1.192	5.561	8.807	1.463	1.597
	Maximum amplitude	3.480	1.302	10.005	1.535	5.828	1.664	9.161	1.534
**C4**	Density	7.065	1.192	3.534	9.249	7.520	0.788	6.093	9.116
	Duration	1.422	0.474	0.816	3.189	1.134	0.137	0.866	1.428
	Mean amplitude	3.398	1.444	1.147	9.548	5.617	1.382	3.409	9.164
	Maximum amplitude	3.611	1.514	1.272	10.114	5.885	1.448	3.543	9.508
**F3**	Density	7.101	0.948	4.174	9.055	6.656	0.823	4.576	8.173
	Duration	1.471	0.467	0.867	3.151	1.056	0.116	0.835	1.319
	Mean amplitude	4.375	1.857	1.618	11.432	4.969	1.401	2.020	9.343
	Maximum amplitude	4.646	1.931	1.760	12.091	5.223	1.454	2.128	9.664
**F4**	Density	7.160	0.930	4.404	9.114	6.682	0.764	5.090	8.063
	Duration	1.474	0.464	0.873	3.148	1.059	0.118	0.831	1.303
	Mean amplitude	4.452	1.891	1.628	12.402	5.006	1.353	2.676	9.263
	Maximum amplitude	4.723	1.965	1.798	13.146	5.256	1.401	2.791	9.588
**O1**	Density	6.931	1.693	1.517	10.019	7.517	0.895	5.352	9.110
	Duration	1.380	0.496	0.757	3.238	1.140	0.140	0.856	1.407
	Mean amplitude	2.142	0.998	0.591	6.085	3.935	1.233	1.877	7.433
	Maximum amplitude	2.317	1.088	0.701	6.818	4.134	1.291	1.978	7.789
**O2**	Density	6.927	1.660	1.540	10.034	7.549	0.901	5.483	9.024
	Duration	1.382	0.498	0.788	3.239	1.138	0.137	0.855	1.428
	Mean amplitude	2.184	1.032	0.604	6.096	3.925	1.157	2.110	7.722
	Maximum amplitude	2.364	1.133	0.703	6.695	4.127	1.211	2.218	8.121
